# Mitochondrial Maturation in Human Pluripotent Stem Cell Derived Cardiomyocytes

**DOI:** 10.1155/2017/5153625

**Published:** 2017-02-27

**Authors:** Dao-Fu Dai, Maria Elena Danoviz, Brian Wiczer, Michael A. Laflamme, Rong Tian

**Affiliations:** ^1^Mitochondria and Metabolism Center, Department of Anesthesiology & Pain Medicine, University of Washington, Seattle, WA, USA; ^2^Department of Pathology, University of Washington, Seattle, WA, USA; ^3^Toronto General Research Institute, McEwen Centre for Regenerative Medicine, University Health Network, Toronto, ON, Canada

## Abstract

Human pluripotent stem cells derived cardiomyocytes (PSC-CMs) have been widely used for disease modeling, drug safety screening, and preclinical cell therapy to regenerate myocardium. Most studies have utilized PSC-CM grown in vitro for a relatively short period after differentiation. These PSC-CMs demonstrated structural, electrophysiological, and mechanical features of primitive cardiomyocytes. A few studies have extended in vitro PSC-CM culture time and reported improved maturation of structural and electromechanical properties. The degree of mitochondrial maturation, however, remains unclear. This study characterized the development of mitochondria during prolonged in vitro culture. PSC-CM demonstrated an improved mitochondrial maturation with prolonged culture, in terms of increased mitochondrial relative abundance, enhanced membrane potential, and increased activity of several mitochondrial respiratory complexes. These are in parallel with the maturation of other cellular components. However, the maturation of mitochondria in PSC-CMs grown for extended in vitro culture exhibits suboptimal maturation when compared with the maturation of mitochondria observed in the human fetal heart during similar time interval.

## 1. Introduction

Human pluripotent stem cells (PSCs) have been widely utilized to generate human cardiomyocytes in vitro for various applications, including disease modeling (disease in a dish model), drug screening, and novel drug discovery, as well as preclinical research for cardiac regeneration in models of myocardial infarction and chronic heart failure [1]. Several methods have been tested and refined to enhance the differentiation efficiency of PSCs into cardiomyocytes, some of which reported the ability to achieve cardiomyocytes population exceeding 90% [2]. These protocols typically involve the sequential application to PSCs of factors including activin A, bone morphogenetic protein-4 (BMP-4), inhibitors or activators of Wnt signaling, and matricellular cues (see recent review [1]).

Regardless of the differentiation method, most studies have cultured PSCs-derived cardiomyocytes (PSC-CM) in vitro for relatively short periods, typically <3–5 weeks after differentiation. The resultant PSC-CMs have underdeveloped electromechanical properties, likely due to the lack of electrophysiological and contractile machinery found in mature or adult cardiomyocytes [3–5]. Recognizing the limitation of using immature PSC-CM for the above applications, we and others have grown in vitro PSC-CM culture for prolonged periods of time, approximately 12–15 weeks, and reported that these late stage PSC-CMs exhibit significantly greater maturation toward an adult cardiomyocyte phenotypes in terms of structural, electrophysiological, and mechanical properties [6–8].

The contractile function of cardiomyocytes is highly energy dependent. As such, maturation of the fetal heart to the adult phenotype is accompanied by a tremendous increase in mitochondrial content via mitochondrial biogenesis, with mitochondrial volume eventually expanding up to ~30% of total cell volume in the adult ventricular myocyte [9]. At the same time, the source of energy production also switches from glycolysis in the fetal heart to predominantly fatty acid oxidation in the adult cardiomyocytes [10, 11]. It is thus likely that PSC-CM with improved mitochondrial maturation would serve as a better “disease in a dish” model for adult cardiomyopathy. Moreover, mitochondrial maturation could bolster myocardial energetics in PSC-CMs and enhance the ability of force generation, which is important for the utility of the PSC derived CM in cardiac regeneration and tissue engineering.

A few studies have reported mitochondrial development and expansion during early differentiation of stem cells into cardiomyocytes [12, 13]. However, limited data is available on the characteristics of mitochondria during the maturation of PSC-CM in vitro. In this study, we assessed mitochondrial abundance, ultrastructure, membrane potential, and respiratory complex activity during maturation of PSC-CM in culture for over 100 days. We report that there is improved mitochondrial maturation with prolonged culture of PSC-CM in vitro, which is in parallel with the maturation of other cellular components. Such progress, however, still lags behind the maturation of mitochondria observed in the fetal heart over a similar time frame.

## 2. Materials and Methods

### 2.1. PSC Maintenance and Cardiomyocyte Differentiation

All experiments were approved by the University of Washington Embryonic Stem Cell Oversight Committee (ESCRO) and conducted using the mT/mG 2APuro RuES-2 hESC line [14] and the IMR90 hiPSC line. PSCs were maintained and differentiated as previously described [6]. Briefly, PSCs were cultured in Matrigel-coated plates (BD Biosciences) and fed with MEF-conditioned media supplemented with 4 ng/mL bFGF (for RuES2) or mTeSR (for IMR90). For cardiomyocytes differentiation, PSC were passaged using versene–EDTA and replated at a density of 150,000–200,000 cells/cm^2^. To start differentiation, we replaced the mTeSR with RPMI-B27 (Gibco) supplemented with L-glutamine, Matrigel, and 100 ng/mL recombinant human activin A (R&D Systems) [2]. After 24 h, the medium was switched to RPMI-B27 supplemented with L-glutamine and 10 ng/mL recombinant human bone morphogenetic protein-4 (BMP-4; R&D Systems). Four days later, the medium was aspirated, and the cells were subsequently fed every other day with RPMI-B27 containing L-glutamine. Cells typically began beating spontaneously on approximately day 14 after differentiation.

### 2.2. hESC- and hiPSC-Cardiomyocyte Maturation

After 20 days of in vitro differentiation, PSC-CMs were replated at 30,000–50,000 cells/cm^2^ onto the PEI-gelatin coated, glass-bottom 35 mm dishes. Cultures were fed every other day thereafter with serum-free RPMI-B27 + L-glutamine. Only cell preparations containing >90% CMs were used for these experiments, and any cultures exhibiting non-CM cell overgrowth were not used. Cultures were monitored for signs of contractile structure organization and the emergence of sarcomere banding.

### 2.3. Purification for hESC-CM

**Figure 1:**
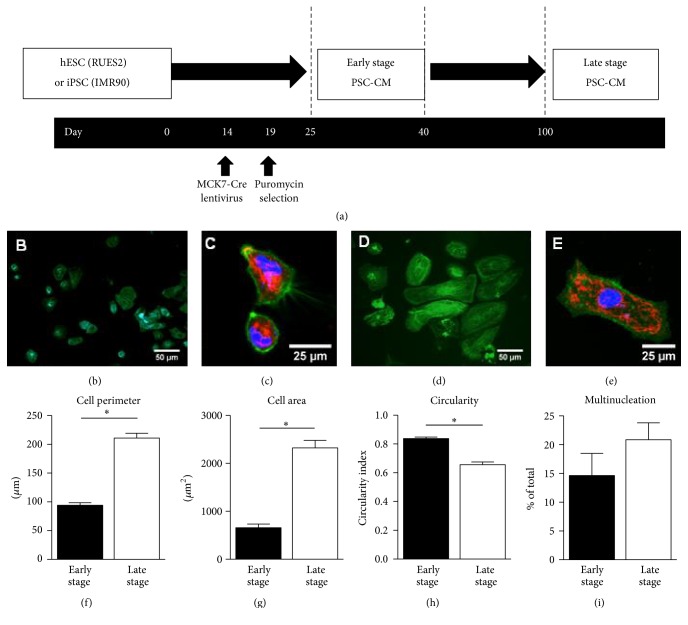
(a) Differentiation and maturation scheme for early and late stage human ESC-CMs (RuES2) and iPSC-CM (IMR90). hESCs were maintained in a mouse embryonic fibroblast-conditioned medium (MEF-CM) supplemented with fibroblast growth factor (FGF). iPSCs were maintained in mTeSR. Both RuES2 and IMR90 stem cells were differentiated into CMs (day 0) by serial treatment with activin A and BMP-4. On day 14, hESC-CMs were transduced with MCK7-Cre lentivirus during replating onto PEI-gelatin coated glass coverslips/plastic plates. On day 19 cells were replated a second time in the presence of 2 ug/mL puromycin and selected for 48 hours. Cells were maintained for approximately 3 months in culture. We classified cells between days 25 and 40 as early stage PSC-CMs and cells > day 100 as late stage PSC-CMs. Alpha-actinin immunofluorescence (b and d) and mitochondrial staining (red)/eGFP (green) (c and e) of early stage (b-c) and late stage (d-e) hESC-CMs. (f–i) hESC-CM morphometric analysis. ^*∗*^*p* < 0.05 relative to early stage hESC-CMs, *n* = 106–121 cells.

To obtain highly purified hPSC-CM for some analysis, we applied mT/mG 2APuro RuES-2 hESC line, which has targeted genomic integration of selectable floxed dual fluorescence reporter (Figure 1), as previously described [14]. These cells originally have cherry tomato fluorescence. After differentiation into hESC-CM expressing MCK7, the MCK7 promoter driven Cre-recombinase will excise the cherry tomato and the hESC-CM will have EGFP fluorescence (Figure 1). These hESC-CM are further selected using puromycin, to get rid of cells not expressing MCK7-driven puromycin resistance genes.

### 2.4. Live Staining with Mitochondrial Markers

Undifferentiated stem cells and human PSC-CMs were plated on fluorodish at least 72 hours before the experiment. For live staining, the culture media in the fluorodish were exchanged with prewarmed (37°C) culture media containing MitoTracker® Green FM (MTG, 25 nM) and Tetramethylrhodamine methyl ester (TMRM, 20 nM), incubated for ~60 minutes, washed 3 times with warm PBS, and then loaded with Hoechst 33342. The cells were imaged using Leica confocal microscope TCS PS8.

### 2.5. Immunofluorescence Staining

Cells were fixed in 4% paraformaldehyde for 10 minutes and permeabilized using 0.25% Triton X-100 for 6 minutes. The fixed cells were blocked with 1.5% normal goat serum for 1 hour at room temperature and incubated overnight at 4°C with a primary antibody at 1 : 500. Antibodies used in this study include mouse anti-*α*-actinin (Sigma), rabbit anti-cTnT (Abcam 91605), rabbit anti-Complex I Ndufs4 (Abcam), mouse anti-Complex II subunit 30 kDa (Invitrogen 459230), rabbit anti-Complex III UQRCR2 (Abcam), and mouse anti-Complex IV subunit II (Invitrogen A-6404). Samples receiving actin staining were incubated with 1 : 50 FITC-labeled phalloidin (Sigma) for 5 min at RT. The samples were rinsed with PBS and incubated with a secondary antibody (Alexa Fluor 488 or 594) at 1 : 500 for 1 h at RT, rinsed, and cover-slipped using Vectashield containing DAPI (Vector Labs).

### 2.6. Transmission Electron Microscopy

Cells were fixed in 1/2 strength Karnovsky's (2% paraformaldehyde/2.5% glutaraldehyde buffered with 0.2 M cacodylate) and postfixed in 2% OsO4 buffered in 0.2 M cacodylate buffer. After quick dehydration, cells were embedded in Epon 812 (Electron Microscopy Sciences), thin sectioned (70 nm), and stained with uranyl acetate for 2 hours and lead citrate for 5 minutes. The samples were imaged using a JOEL 1230 transmission electron microscope set to 80 kV, and the images were captured using a Gatan digital imaging system. Image analysis was performed using NIH Image J, for mitochondrial size and relative abundance. The latter was calculated by fraction of mitochondria area/cell area.

### 2.7. Imaging and Morphological Analysis

Bright-field and fluorescent images were acquired using a Leica TCS SP8 confocal microscope or Nikon fluorescence microscope. Images were processed using the Leica LAS-X software; the images were quantified in Image J software, using standard analysis plugins. Cells were analyzed for cell area, cell perimeter, and cell circularity index. Quantitative analysis was performed on 15–20 fields from three replicates.

### 2.8. Mitochondrial Respiratory Complex Activity Assays

Human fetal hearts (50–60 or 100–115 days gestational age) were obtained from the University of Washington Birth Defects Research Laboratory, and the adult heart samples were obtained from donor hearts unsuitable for transplant. The University of Washington Institutional Review Board (IRB) for Human Subjects determined that these anonymous human biological materials were derived from otherwise discarded tissue and thus would not be considered human subjects research (IRB Determination # 08-0062-N).

All samples (tissue or cells) were previously frozen and thawed right before the assays. For PSC-CMs, lysates from ~100,000 cells were used to determine the activity of each complex, while, for human fetal and adult heart tissue, a total of 30 *μ*g protein from tissue lysate was used. Assessment of respiratory complex activities was performed using the three spectrophotometric assay methods described previously [15], with modifications. Briefly, Complex I activity was measured at 340 nm using 100 *μ*M decylubiquinone as an acceptor and 250 *μ*M NADH as a donor, in 10 mM Tris-HCl buffer (pH 8.0) containing 1 mg/mL of BSA and 300 *μ*M KCN for ~2 min. The addition of 8 *μ*M rotenone allowed the quantification of the rotenone-sensitive Complex I activity. Complex II activity was measured at 600 nm using 40 *μ*M DCPIP, 80 *μ*M decylubiquinone, 500 *μ*M ATP, and 8 *μ*M rotenone in a buffer containing 10 mM potassium phosphate (pH 7.2), 1 mg/mL of BSA, and 300 *μ*M KCN for 3 min, preincubated at 37°C. The reaction was initiated by adding 5 mM succinate and then recorded for 2 minutes. The oxidation of succinate was inhibited by 5 mM malonate. Complex III activity was measured at 550 nm using 50 *μ*M oxidized cytochrome* c* as an acceptor and 50 *μ*M decylubiquinol as a donor in a buffer containing 10 mM potassium phosphate (pH 7.2), 300 *μ*M KCN, and 2 mM EDTA. After the addition of 10 *μ*g/mL of antimycin A, Complex III specific activity corresponding to the antimycin A-sensitive activity was measured for 1 minute. Complex IV activity was measured at 550 nm in a buffer containing 10 mM potassium phosphate (pH 7.2), 1 mg/mL BSA, and the cell/tissue lysate. The reaction was initiated by adding 10 *μ*M reduced cytochrome* c*. The decrease in absorbance was observed for 2 minutes. KCN (300 *μ*M) was applied to check the specificity of Complex IV activity. The citrate synthase assay was measured at 412 nm for the reduction of 100 *μ*M DTNB in the presence of 300 *μ*M acetyl-CoA in 100 mM Tris (pH 8.0) and 0.1% (vol/vol) Triton X-100 medium. The reaction was initiated by adding 500 *μ*M oxaloacetic acid and measured for 3 min.

### 2.9. Statistical Analysis

Pairwise comparisons were performed using Student's *t*-test, while multiple comparisons were performed using one-way ANOVA with a Sidak post hoc test to determine statistical significance. All data is reported as the mean ± standard error of the mean (SEM). Statistically significant differences were defined as *p* < 0.05. All statistical analyses were done with Stata Intercooled Version.

## 3. Results

Our experimental design is depicted in Figure 1(a). In brief, human PSC-CMs were generated using a previously reported cardiac differentiation protocol involving the serial application of activin A and BMP-4 to PSCs maintained in monolayer cultures on Matrigel-coated plates [2, 6]. To achieve highly purified populations of hPSC-CMs, we employed a genetic selection strategy based on a previously described transgenic hESC line suitable for Cre-lox-mediated fate mapping [14]. In brief, we used the RuES2 hESCs that had been stably engineered with a floxed “stoplight” fluorescent reporter system (mTmG-2A Puro transgene) in which expression of Cre-recombinase mediates a switch from constitutive expression of membranous cherry tomato red fluorescent protein to constitutive expression of green fluorescent protein (GFP) and puromycin resistance. To enable selection of cardiomyocytes, mTmG-2A Puro-RuES2 hESC-CMs after ~14 days of in vitro differentiation were transduced with a lentiviral vector in which the striated muscle-specific muscle creatine kinase-7 (MCK-7) regulator cassette drives expression of Cre-recombinase [16–18]. Transduced cultures were treated with puromycin for 5 days, resulting in 99% GFP+ cardiomyocytes as previously reported. The resultant genetically selected hESC-CMs were then maintained on polyethyleneimine and gelatin coated plates prior to analysis at one of two time-points as described in earlier work by our laboratory: “early stage” cultures (i.e., after 25–40 days of in vitro differentiation) or “late stage” cultures (i.e., after >100 days of in vitro differentiation) [6]. Early and late stage hESC-CM were processed for immunofluorescence staining and then analyzed for cell morphometry (Figures 1(b)–1(e)) and collected for mitochondrial respiratory activity. When compared with early stage hESC-CM, late stage hESC-CM had significantly longer cell perimeter, Figure 1(f), larger cell area, Figure 1(g), less circularity or more elongation, Figure 1(h), and a nonsignificant increase in the percentage of multinucleated cells, Figure 1(i). These findings are consistent with previous report from our laboratory [6].

**Figure 2:**
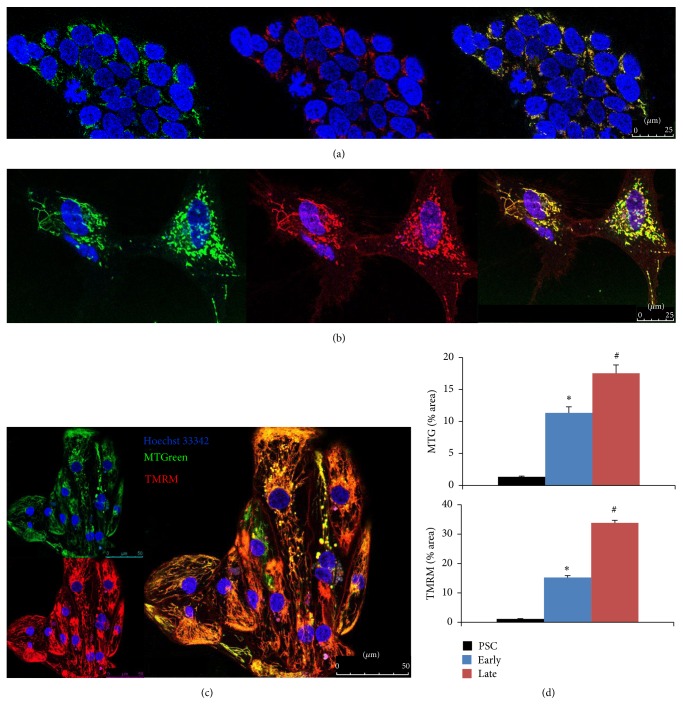
Live staining of representative undifferentiated hPSC (a), early hPSC-CM (b), and late hPSC-CM (c) with MitoTracker Green (MTG), TMRM (red), and Hoechst 33342 (blue) and the overlay images. (d) Quantitative image analysis of relative area of MTG (above) and TMRM (bottom) staining. ^*∗*^*p* < 0.001 compared with PSC, ^#^*p* < 0.001 compared with early stage hPSC-CMs.

To test the hypotheses that these structural changes would be accompanied by similar level of mitochondrial maturation, we performed live staining of undifferentiated hESC (mTmG RuES2) and hESC-CM at early and late stages with two mitochondrial indicators, TMRM and MitoTracker Green FM (MTG). TMRM has been used as an indicator of intact mitochondrial membrane potential, and MTG has been used to localize mitochondria independent of mitochondrial membrane potential. As shown in Figures 2(a) and 2(b), there was a dramatic increase in the mitochondrial fluorescence staining from undifferentiated hESC to early hESC-CM, indicating increased abundance of mitochondria when cells differentiated into early cardiomyocytes. While the change in percentage areas of mitochondrial staining indicated by MTG from early, Figure 2(b), to late stage hESC-CM, Figure 2(c), was modest, there was a prominent increase in TMRM (red) from early to late stage hESC-CM, suggesting a maturation of mitochondrial membrane potential seen at late stage hESC-CM. This observations were confirmed by quantitative image analysis (Figure 2(d)), which showed significant increase in relative areas of MTG and TMRM staining during hESC-CM differentiation and maturation. Of note, the increase in relative area of TMRM is more prominent from early to late stage of PSC-CMs.

**Figure 3:**
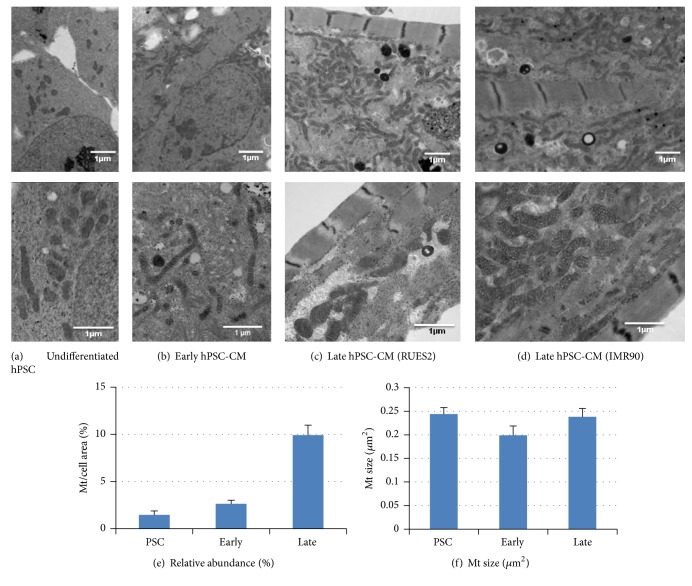
EM images of undifferentiated RuES2 hPSC (a), early stage RuES2 PSC-CM (b), late stage RuES2 PSC-CM (c), and late stage IMR90 iPSC-CM (d) at lower (upper panel) and higher magnification (lower panel). Quantitative analysis of relative abundance of mitochondria (e, calculated by mitochondria area/cell area) and mitochondrial size (f). Data represented as mean ± SEM, *n* = 321 for early stage and *n* = 433 for late stage. Data represented as mean ± SEM. For each condition 16 fields were examined.

Transmission electron microscope examination was performed to determine the relative amount and size of mitochondria during the differentiation and maturation of hESC-CMs (Figure 3). There was ~2-fold increase in mitochondrial relative abundance during differentiation (*p* < 0.01 for undifferentiated hESC versus early hESC-CMs, Figures 3(a), 3(b), and 3(e)) and ~2.8-fold increase in mitochondrial relative abundance during maturation from early to late hESC-CM (*p* < 0.01, Figures 3(b)–3(e)). Higher magnification electron micrographs (lower panel of Figures 3(a)–3(d)) revealed that the size of mitochondria did not change significantly during differentiation and maturation, confirmed by quantitative analysis (Figure 3(f)). We also investigated the maturation of mitochondria using human induced pluripotent stem cells derived cardiomyocytes (hiPSC-CM) of IMR90 line (Figure 3(d)) and found similar mitochondrial maturation comparable to that seen in hESC-CM (Figure 3(c)).

**Figure 4:**
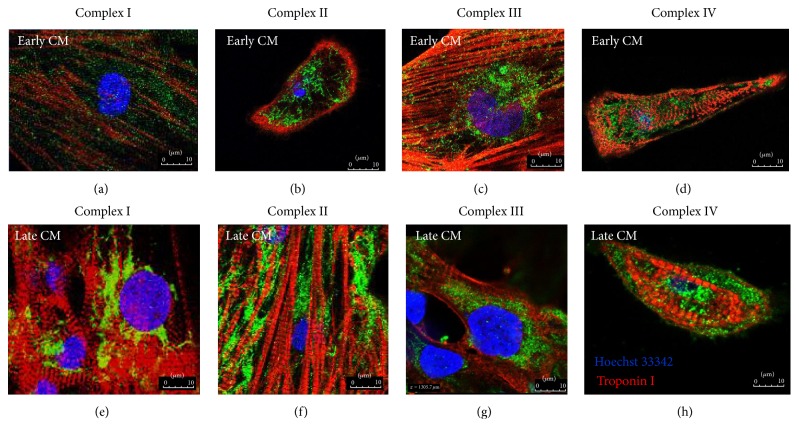
Immunofluorescence staining of mitochondrial respiratory Complexes I–IV (green), Troponin I (red), and Hoechst 33342 (blue) in early hPSC-CM (a–d) and late hPSC-CM (e–h).

**Figure 5:**
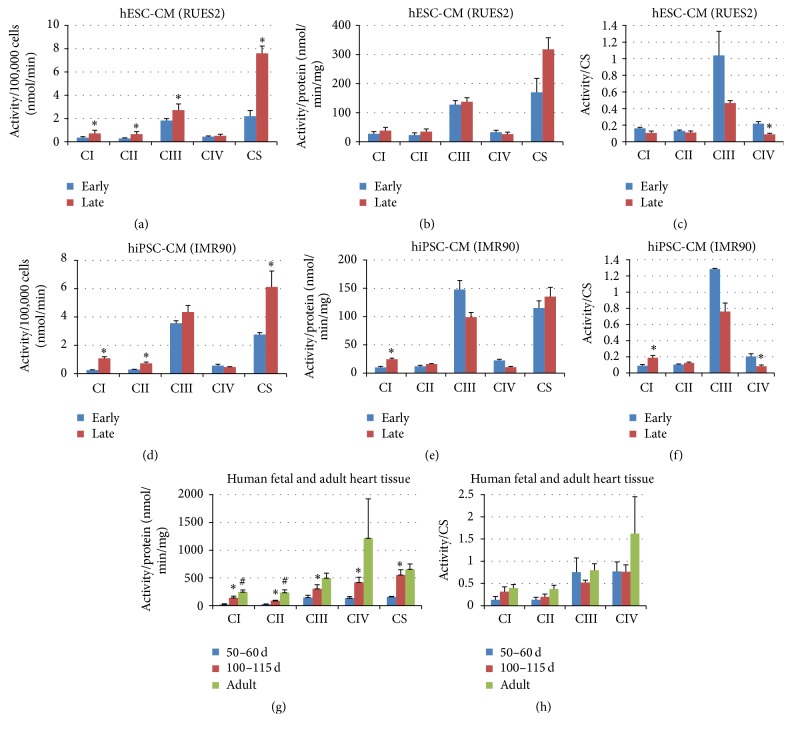
Respiratory complex activities in hESC-CM (a–c), hiPSC-CM (d–f), and human heart tissue (g, h). Respiratory complex activities are measured in 100,000 cells of either hESC-CM (hESC-CM) or hiPSC-CM (d), normalized to total protein (b, e) or citrate synthase activity (c, f). Respiratory complex activities are measured in fetal hearts (50–60 days and 100–115 days), and adult heart tissue lysate, normalized to protein amount (g) or citrate synthase activity (h) of the corresponding samples. ^*∗*^*p* < 0.05 versus early CM or 50–60 d CM. ^#^*p* < 0.05 adult versus 100–115 d; *n* = 3–5 biological replicates.

Since the generation of mitochondrial membrane potential depends on the maturation of electron transport chains (ETC), we performed immunofluorescence staining for Complexes I–IV in early versus late hPSC-CM. As shown in Figure 4, the staining for mitochondrial electron transport Complexes I–IV (green) increased in area and intensity during maturation. The increase appeared to be more prominent for Complexes I and II (Figures 4(a), 4(e), 4(b), and 4(f)). Changes in the ETC appeared to be in synchrony with better development of sarcomeres (red), evidenced by more abundant and better organization of parallel array. For better quantification of mitochondrial electron transport chain during maturation from early to late cultures, we performed respiratory complex activity assay in cell lysate of both hESC-CM (RuES2) and hiPSC-CM (IMR90) using a spectrophotometric method (Figure 5). These activity assays were normalized to the number of differentiating cardiomyocytes (per 100,000 cells, Figures 5(a) and 5(d)), total protein extract of these cardiomyocytes (Figures 5(b) and 5(e)), and citrate synthase activity of the same samples (Figures 5(c) and 5(f)). As shown in Figure 5(a), there was a significant increase in respiratory complex activity per 100,000 cells for Complexes I, II, and III and citrate synthase from early to late RuES2-CM. This was closely recapitulated in IMR-90-CM for Complexes I and II and citrate synthase (Figure 5(d)). The increase in complex activity on a per cell basis was likely due to the increase of cell size during maturation of cardiomyocytes. The difference between the early and late stage cardiomyocytes diminished when the complex activity was normalized to the total protein amount (Figures 5(b) and 5(e)). Likewise, there is no increase in relative Complexes I–IV activity when normalized to citrate synthase activity, an indicator of mitochondrial mass, suggesting that the complex activity on the per mitochondrion basis did not change during prolonged culture. Indeed, relative Complex IV activity (normalized to citrate synthase) significantly declined from early to late cardiomyocytes (Figures 5(c) and 5(f)). Collectively, these data suggest that the increase of cell size during the prolonged culture was matched by the increases of mitochondrial and nonmitochondrial proteins. We did not observe any mitochondrial maturation disproportionate to overall cell growth.

To compare the development of mitochondria in vitro in hPSC-CM with in vivo human fetal heart, we performed similar mitochondrial activity assay using fetal heart extracts obtained at 50+ d (50–60 days) or 100+ d (100–115 days) gestational ages. The complex activities measured in adult human heart extracts (from deceased donor hearts with normal cardiac function) were used as a reference. As shown in Figure 5(g), the respiratory complex activities normalized to total protein significantly increased for all Complexes I–IV from 50+ d to 100+ d in fetal hearts. Further increases from 100+ d fetal to adult hearts were moderate and only significant for Complexes I and II. The increase was not obvious when complex activity was normalized to citrate synthase (Figure 5(h)). These results suggest that a surge of mitochondrial growth over other cellular components occurred during 50 to 100 days in the fetal heart, which we did not observe in cultured PSC-CMs.

## 4. Discussion

In this study, we demonstrate that differentiation of PSC into cardiomyocytes is associated with substantial mitochondrial biogenesis, which continues during the maturation of cardiomyocytes in culture. A significant increase of mitochondrial abundance can be achieved by extending the in vitro culture from 30 days to 100 days. However, distinct from fetal development, the relative mitochondrial enzyme activity in PSC-CM normalized to total protein or citrate synthase activity remains unchanged during prolonged culture, despite the maturation in other parameters obtained by prolonged in vitro culture. These findings indicate inadequate maturation of mitochondria despite increase in relative abundance or mass, even after prolonged in vitro culture.

Most studies using PSC-CMs for disease modeling or for preclinical cardiac cell therapy have applied cells differentiated in vitro for relatively short period of time, approximately 2–5 weeks after differentiation, when these PSC-CMs demonstrate some phenotypes of early cardiomyocytes, such as troponin genes expression and early myofibrillar bundles forming Z-bands similar to early fetal cardiomyocytes [19]. These early and immature PSC-CMs display primitive calcium transients and electrophysiological properties. Furthermore, the capacity of mechanical contraction is rather limited [3–5, 20]. Cardiac cell therapy using these early immature PSC-CM have shown mixed results, which is expected based on limited contribution of these early PSC-CMs on force generations. Although some studies did show improvement of cardiac function, these injected PSC-CMs need to have enhanced mitochondrial function to match maturation in electrophysiological and contractile function.

Although several methods have been explored to stimulate cardiomyocyte maturation, however, the most straightforward method is to extend the in vitro culture time. Prolonged culture has been shown to enhance structural organization and functional performance over time. Previous study from our group has reported that late stage PSC-CMs grown in vitro for more than 100 days exhibit improved functional performance as measured by contractile, calcium-transient, and electrophysiological properties, to the levels approaching those found in adult CMs. Prolonged in vitro culture of PSC-CMs has been reported to gain ~2-fold enhancement of contraction and improved Ca-transients, compared with early PSC-CMs [4, 6]. These late stage PSC-CMs are expected to have greater capacity to generate new force-producing units and perhaps improve heart failure upon transplantation.

One critical aspect of PSC-CMs that has not been fully explored is the maturation of their mitochondria. Adult cardiomyocytes are highly enriched in mitochondria, and their electromechanical activity is highly dependent on ATP generated by oxidative phosphorylation. Mitochondrial dysfunction has been widely documented in congestive heart failure in human patients and several experiment models of cardiomyopathy [21]. Consistent with this, mitochondrial oxidative damage has been shown to mediate the phenotypes of cardiomyopathy in various mouse models [22, 23]. Therefore, it is expected that using PSC-CMs with immature mitochondria in disease modeling or drug screening may interfere with the characterization of cardiomyopathy phenotype. Thus, achieving optimal mitochondrial maturation is essential for successful application of PSC-CMs.

In this study, we demonstrate some degree of mitochondrial maturation with prolonged duration of in vitro culture exceeding 100 days. This maturation includes 2.8-fold increase in relative abundance (Figures 2 and 3), increased enzyme amount (Figure 4), and activity of respiratory Complexes I–III as well as citrate synthase (Figures 5(a)–5(f)). The size of mitochondria does not appear to change from early to late PSC-CMs; however, there is an increase in TMRM staining, suggestive of enhanced mitochondrial membrane potential (Figure 2).

To compare the effect of extended in vitro culture of PSC-CM and in vivo fetal heart development, we perform respiratory complex activity assay for total heart extracts from equivalent gestational age of 50+ d (50–60 days) and 100+ d (100–115 days). Fetal heart tissues contain significant fractions of noncardiomyocytes, such as fibroblasts and endothelial cells, and hence are not directly comparable to the protein extracts from PSC-CMs; however, comparisons between 50+ d to 100+ d fetal heart development and early to late PSC-CM maturation are valid. Normalized to the total protein, the activity of respiratory complex from 50+ d to 100+ d fetal hearts significantly increases by 6.4-fold for Complex I, by 3.6-fold for Complex II, by 2.1-fold for Complex III, by 3.1-fold for Complex IV, and by 3.5-fold for citrate synthase. This increase in respiratory complex activity during fetal heart development is at least a few times higher than that seen in PSC-CM culture, indicating inadequate maturation of mitochondria in prolonged in vitro culture. The respiratory complex activity (normalized to total protein) of adult heart tissue extract is further increased compared to 100-day fetal heart, reaching 3.4- to 11.3-fold over those in 50+ d fetal hearts, demonstrating an impressive magnitude of mitochondrial maturation during fetal and postnatal development.

Inadequate maturation remains one of the major hurdles in application of these cells. Extending the in vitro culture beyond 100 days is a very slow process and even late stage cardiomyocytes do not fully recapitulate the adult cardiomyocyte phenotype. Notably, mitochondrial abundance by area is ~10% greater than 100 days after differentiation, which is far below the approximate 30% observed in adult CM [9]. Furthermore, the rapid maturation of mitochondria relative to other cellular components during fetal heart development mentioned above is not observed in prolonged in vitro culture. Delayed mitochondrial maturation could be attributed to nonphysiological culture conditions, such as the lack of electromechanical stimulation and interactions with other noncardiomyocytes. Previous studies have attempted to address this issue by applying electromechanical conditioning or growing PSC-CM in biomimetic environments: coculture with noncardiomyocytes, three-dimensional culture, or adding specific growth factors [24–27]. Most of these studies have focused on enhancing mechanical and electrophysiological maturations. Yang et al. reported that the addition of T3 thyroid hormone enhanced maximal mitochondrial respiratory capacity and respiratory reserve capacity [24], in parallel with improved electromechanical properties.

## 5. Conclusion

In summary, prolonged in vitro culture of PSC-CM demonstrates some maturation of mitochondria, yet it is suboptimal compared with in vivo fetal heart maturation. Further studies are needed to optimize and enhance mitochondrial maturation, such as growing PSC-CM in better biomimetic environment for longer period of time, in order to obtain reliable PSC-CM cells for cardiomyopathy modeling and cardiac cell therapy.
